# On the linear programming bound for linear Lee codes

**DOI:** 10.1186/s40064-016-1863-8

**Published:** 2016-03-01

**Authors:** Helena Astola, Ioan Tabus

**Affiliations:** Department of Signal Processing, Tampere University of Technology, 33101 Tampere, Finland

**Keywords:** Lee codes, Linear codes, Linear programming bound, Lee-compositions, Lee-numbers

## Abstract

Based on an invariance-type property of the Lee-compositions of a *linear* Lee code, additional equality constraints can be introduced to the linear programming problem of linear Lee codes. In this paper, we formulate this property in terms of an action of the multiplicative group of the field ${\mathbb {F}}_{q}$ on the set of Lee-compositions. We show some useful properties of certain sums of Lee-numbers, which are the eigenvalues of the Lee association scheme, appearing in the linear programming problem of linear Lee codes. Using the additional equality constraints, we formulate the linear programming problem of linear Lee codes in a very compact form, leading to a fast execution, which allows to efficiently compute the bounds for large parameter values of the linear codes.

## Background

Finding the largest code (in cardinality) with a given length and minimum distance is one of the most fundamental problems in coding theory. The most well-known upper bounds for the Hamming metric are the Hamming bound, Plotkin bound, Singleton bound and Elias bound, and these bounds have been formulated for the Lee metric also, although the expressions are slightly more complicated. Delsarte (1973) introduced association schemes to coding theory to deal with topics involving the inner distribution of a code. An important approach arises from association schemes to the problem of determining the upper bound for the size of a code, namely the linear programming approach. The asymptotically best upper bound in the Hamming metric is the McEliece–Rodemich–Rumsey–Welch bound, see McEliece et al. (1977), which is based on the linear programming approach, and gives a substantial improvement to the earlier best upper bound, which is the Elias bound. In the Hamming metric, the distance relations between codewords directly define an association scheme, but in the Lee metric, the distance relations between the Lee-compositions define the association scheme. The Lee association scheme and linear programming bounds for Lee codes have been discussed by Astola (1982a), Solé (1988), and Tarnanen (1982). Generalizing to finite Frobenius rings, the linear programming bound for codes equipped with homogeneous weight, including the Lee weight on ${{\mathbb {Z}}}_{4}$, has been studied by Byrne et al. (2007).

We denote ${\mathbb {Z}}_q^n = \{{\mathbf {x}} = [x_1,\ldots ,x_n]| x_i\in \{0,1,\ldots ,q-1\}\}$ the set of length *n* vectors having $q-$ary elements. The Hamming distance between two vectors ${\mathbf {x}}, {\mathbf {y}}\in {\mathbb {Z}}_q^n$ is $d_H({\mathbf {x}}, {\mathbf {y}}) = |\{i \mid x_{i}\ne y_{i}\}|$, and penalizes equally any non-zero error between the components of $\mathbf {x}$ and $\mathbf {y}$, being a natural measure for errors in many communication channels. However, in communication channels with phase modulation, where the symbols $0,1,\ldots ,q-1$ are transmitted as phases $0,{2\pi \over q},\ldots ,(q-1){2\pi \over q}$, the errors are measured as the shortest distance between the symbols along the unit circle and hence we consider in the following the error between two symbols $i,j\in {\mathbb {Z}}_q$ to be defined as the Lee distance $d_L(i,j) = \min (|i-j|,q-|i-j|)$, which was introduced by Lee (1958). The Lee distance between two vectors ${\mathbf {x}}, {\mathbf {y}}\in {\mathbb {Z}}_q^n$ is defined as

$$\begin{aligned} d_L ({\mathbf {x}},{\mathbf {y}}) = \sum _{i=1}^n \min \left( |x_i-y_i|, q-|x_i-y_i|\right) . \end{aligned}$$

The Lee weight of one symbol $j\in {\mathbb {Z}}_q$ is $w_L(j) = \min (j,q-j)$ and that of one vector ${\mathbf {x}}\in {\mathbb {Z}}_q^n$ is $w_L({\mathbf {x}}) = d_L({\mathbf {0}},{\mathbf {x}})$. The number of components of the vector ${\mathbf {x}}\in {{\mathbb {Z}}}_q^n$ having the Lee weight equal to *i* is denoted $l_i(\mathbf {x})$ and, since $l_i({\mathbf {x}}) = l_{q-i}({\mathbf {x}})$, only $s= \lfloor {q\over 2}\rfloor$ such numbers describe completely the distribution of the Lee weights of the elements of $\mathbf {x}$. This distribution, denoted $l(\mathbf {x})$, is called the Lee-composition of $\mathbf {x}$, defined as:

$$\begin{aligned} l({\mathbf {x}}) = \left[ l_0({\mathbf {x}}), l_1({\mathbf {x}}), \ldots , l_s({\mathbf {x}}) \right] . \end{aligned}$$

We denote ${\mathcal{L}}_q^n = \{{\mathbf {t}}_0,\ldots ,{\mathbf {t}}_{\alpha }\} = \{l({\mathbf {x}})| {\mathbf {x}} \in {\mathbb {Z}}_q^n \}$ the set of distinct Lee-compositions, where $(\alpha +1)$ is their number, and we reserve the notation ${\mathbf {t}}_0$ for $l({\mathbf {0}})=[n,0,\ldots ,0]$.

A code is defined as a subset of vectors of ${\mathbb {Z}}_q^n$. Keeping with the communication scenario, only the codewords belonging to the code *C* are sent as messages over the communication channel, say the codeword ${\mathbf {x}}\in C$ is sent and is corrupted due to communication errors, being received as a different vector, ${\mathbf {z}} \in {\mathbb {Z}}_q^n$. If the minimum distance *d* between all codewords of *C* is $2e+1$, then all the spheres $\{{\mathbf {y}}| d_L({\mathbf {x}},{\mathbf {y}}) \le e, {\mathbf {x}} \in C\}$ around each codeword ${\mathbf {x}} \in C$ are non-intersecting, and the received vector ${\mathbf {z}}$ can be correctly decoded if $d_L({\mathbf {z}},{\mathbf {x}}) \le e$. When designing codes *C*, the minimum distance *d* of the code is imposed as an initial requirement. The goal is then to design a code having the largest number of codewords, so that one can send reliably as many messages as possible, with the guarantee of correcting errors of Lee weight smaller than or equal to *e*. Since the problem of designing the largest codes is not solved yet in general, finding upper bounds on the size of the code *C* for a given *d* is considered one of the major problems in information theory. For example when new codes are proposed, comparing their size against the known upper bounds allows to prove their optimality if they achieve the upper bounds.

In the case of Lee distance, the constraints that need to be satisfied by an error correcting code with a given distance *d* can be conveniently analyzed using the association scheme introduced by Delsarte (1973). The scheme has $\alpha +1$ relations denoted $K_{{\mathbf {t}}_0}, \ldots , K_{{\mathbf {t}}_\alpha }$, where $({\mathbf {x}}, {\mathbf {y}})$ belongs to $K_{{\mathbf {t}}_i}$ if $l({\mathbf {x}} -{\mathbf {y}}) = {\mathbf {t}}_i$, with ${\mathbf {x}}, {\mathbf {y}}\in {{\mathbb {Z}}}_q^n$ and ${\mathbf {t}}_i\in {\mathcal{L}}_q^n$.

Introduce the inner distribution of the code *C* as

$$\begin{aligned} B_{{\mathbf {t}}_i} = {1\over |C|} |K_{{\mathbf {t}}_i} \cap C^2|, \ \ \ {\mathbf {t}}_i \in {\mathcal{L}}_q^n, \end{aligned}$$

where |*C*| denotes the cardinality of the set *C*, and in particular $B_{{\mathbf {t}}_0}=1$. Hence the desired quantity to be optimized for a code, its cardinality |*C*|, can be simply expressed in terms of the variables $B_{{\mathbf {t}}_i}$ as

$$\begin{aligned} \sum _{{\mathbf {t}}_i \in {\mathcal{L}}_q^n} B_{{\mathbf {t}}_i} = |C|. \end{aligned}$$

The fundamental result of the association scheme introduced by Delsarte (1973) is the formulation of the linear constraints between the variables $B_{{\mathbf {t}}_i}$, using as coefficients the so-called Lee-numbers, which are the eigenvalues of the Lee association scheme. Let $\xi = exp(2\pi \sqrt{-1}/q)$. The Lee-number $L_{\mathbf {t}}(\mathbf {u})$ with ${\mathbf {t}},{\mathbf {u}}\in {\mathcal{L}}_q^n$ can be computed as follows: take any vector $\mathbf {v}$ having the Lee-composition ${\mathbf {u}} = l({\mathbf {v}})$ and compute (see Astola 1982b)

1 $$\begin{aligned} L_{{\mathbf {t}}}({\mathbf {u}})= \sum _{{\mathbf {x}}\mid l({\mathbf {x}})={\mathbf {t}}} \left( \prod _{i=1}^{n} \xi ^{v_{i}x_{i}}\right) . \end{aligned}$$

The linear inequality constraints between the variables $B_{{\mathbf {t}}_i}$ are then expressed as (see Astola 1982b):

$$\begin{aligned} \sum _{i=1}^{\alpha } B_{{\mathbf {t}}_i} L_{\mathbf {k}}({\mathbf {t}}_i) \ge - \left[ \begin{matrix} n\\ {\mathbf {k}} \end{matrix}\right] , \end{aligned}$$

where $\genfrac[]{0.0pt}{}{n}{\mathbf {k}}={n \atopwithdelims (){\mathbf {k}}}2^{n-k_{0}}$ for $q=2s+1$, and ${n \atopwithdelims ()\mathbf {k}}$ is the multinomial coefficient ${n\atopwithdelims ()k_{0},\ldots ,k_{s}}$.

With these constraints, the cardinality $\sum _{{\mathbf {t}}_i \in {\mathcal{L}}_q^n} B_{{{\mathbf {t}}}_i} = |C|$ of any code can be upper bounded by solving a linear programming problem, as stated in the following (see Astola 1982b). Let $B_{\mathbf {t}}^{*}, \ {\mathbf {t}}\in \left\{ {\mathbf {t}}_{1}, \ldots ,{\mathbf {t}}_{\alpha }\right\}$ be an optimal solution of the linear programming problem

2 $$\begin{aligned}&\hbox {maximize}\sum _{i=1}^{\alpha }B_{{\mathbf {t}}_{i}} \\&B_{{\mathbf {t}}_{i}}\ge 0, \quad i\in I \hbox { and }B_{{\mathbf {t}}_{i}}= 0, \quad i\in \{1,\ldots ,\alpha \}{\setminus } I\nonumber \\&\sum _{i=1}^{\alpha } B_{{\mathbf {t}}_{i}} L_{{\mathbf {k}}}({\mathbf {t}}_{i})\ge - \left[ \begin{matrix} n\\ {\mathbf {k}}\end{matrix}\right] , \ \quad \hbox {for all }{\mathbf {k}}\in {\mathcal{L}}_q^n\nonumber \end{aligned}$$

where $I = \{i\mid l({\mathbf {x}})={\mathbf {t}}_{i} \Rightarrow w_{L}({\mathbf {x}})\ge d\}$. Then $1+\sum _{i=1}^{\alpha } B_{{\mathbf {t}}_{i}}^{*}$ is an upper bound to the size of the code *C* with the minimum distance *d*.

We have previously shown that in the case of *linear* Lee codes we can use the following sharpening of the linear programming problem (see Astola and Tabus 2015). Let $B_{\mathbf {t}}^{*}, \ {\mathbf {t}}\in \left\{ {\mathbf {t}}_{1}, \ldots ,{\mathbf {t}}_{\alpha }\right\}$ be an optimal solution of the linear programming problem

3 $$\begin{aligned}&\hbox {maximize}\sum _{i=1}^{\alpha }B_{{\mathbf {t}}_{i}} \\&B_{{\mathbf {t}}_{i}}\ge 0, \quad i\in I \hbox { and }B_{{\mathbf {t}}_{i}}= 0, \quad i\in \{1,\ldots ,\alpha \}{\setminus } I\nonumber \end{aligned}$$

4 $$\begin{aligned}&B_{{\mathbf {t}}_{i}}=B_{{\mathbf {t}}_{j}} \quad \hbox { for all } {\mathbf {t}}_{j}\in \tau ({\mathbf {t}}_{i}) \end{aligned}$$

5 $$\begin{aligned}&\sum _{i=1}^{\alpha } B_{{\mathbf {t}}_{i}} L_{\mathbf {k}}({\mathbf {t}}_{i})\ge - \left[ \begin{matrix} n\\ {\mathbf {k}} \end{matrix}\right] , \ \quad \hbox {for all }{\mathbf {k}}\in {\mathcal{L}}_q^n \end{aligned}$$

where $\tau ({\mathbf {t}}_{i})$ is the set of Lee-compositions that are obtained when the vectors $\mathbf {x}$ having the Lee-composition ${\mathbf {t}}_{i}$ are multiplied by all $r\in {\mathbb {F}}_{q}\backslash \{0\}$, and $I = \{i\mid l({\mathbf {x}})={\mathbf {t}}_{i} \Rightarrow w_{L}({\mathbf {x}})\ge d\}$, i.e., the indices of those Lee-compositions corresponding to vectors of Lee weight $\ge d$. Then $1+\sum _{i=1}^{\alpha } B_{{\mathbf {t}}_{i}}^{*}$ is an upper bound to the size of the code *C* with the minimum distance *d*.

The above sharpening is based on an invariance-type property of the Lee-compositions of a linear code. In the Hamming metric, multiplying codewords by a constant does not change the weight of the codewords. However, in the Lee metric, multiplication typically changes the Lee-composition of the codeword and so also usually the Lee weight. With linear codes, since they are linear subspaces of vector spaces, all the multiplied versions of any codeword also belong to the code. The authors have previously shown that there are as many codewords having the Lee-composition of a given codeword $\mathbf {x}$ as there are codewords having the Lee-composition of $r\mathbf {x}$ that is obtained by multiplication of the given codeword $\mathbf {x}$ by some constant *r* (see Astola and Tabus 2015).

In this paper, we formulate this property of Lee-compositions of a *linear* Lee code in terms of an action of the multiplicative group of the field ${\mathbb {F}}_{q}$ on the set of Lee-compositions. This formulation gives theoretical tools for studying the Lee-compositions and linear Lee-codes. For simplicity, we let *q* be prime, ${\mathbb {F}}_{q}^{n}=\{{\mathbf {x}}\mid x_{i}\in {\mathbb {F}}_{q},i=1,\ldots ,n\}$. In addition, we show some useful properties of certain sums of Lee-numbers. Using the equality constraints introduced by Astola and Tabus (2015) and the properties of certain sums of Lee-numbers, we may compact the set of variables and linear constraints in the linear programming problem, and perform all computations with rational numbers. Compacting the problem leads to a faster execution, which allows to efficiently compute the bounds for large parameter values.

## The group action

In the paper by Astola and Tabus (2015), a mapping $\tau (\mathbf {t})$ that maps the Lee-composition $\mathbf {t}$ into the set of Lee-compositions, which are obtained from $\mathbf {t}$ by multiplication of vectors having the Lee-composition $\mathbf {t}$ by all $r\in \{1,\ldots ,q-1\}$, was defined in the following way:

$$\begin{aligned} \tau ({\mathbf {t}})= & {} \left\{ \left[ t_{\pi _{r}(0)}({\mathbf {x}}), t_{\pi _{r}(1)}({\mathbf {x}}),\ldots ,t_{\pi _{r}(s)}({\mathbf {x}})\right] \mid {\mathbf {t}}=l({\mathbf {x}}),\pi _{r}(i)=|k|, \right. \nonumber \\&\left. kr \equiv i \mod q,-s\le k \le s, \ 1\le r\le q-1 \right\} , \end{aligned}$$

where

$$\begin{aligned} \pi _{r}(i)=|k|\hbox { such that }rk \equiv i \mod q\hbox { and }-s\le k \le s. \end{aligned}$$

The mapping of the Lee-compositions can be formulated in terms of a group action.

### **Definition 1**

If *G* is a group and *X* is a set, then a group action $\varphi$ of *G* on *X* is a function

$$\begin{aligned} \varphi :G\times X\rightarrow X \end{aligned}$$

that satisfies the following conditions for all $x\in X$:

1. $\varphi (e,x) = x$, where *e* is the identity element of *G* (identity).
2. $\varphi (g,\varphi (h,x))=\varphi (gh,x)$ for all $g,h\in G$ (compatibility).

For notational reasons, denote in the following the set of Lee-compositions ${\mathcal{L}}_q^n$ as $\{\rho ^{(0)},\ldots ,\rho ^{(\alpha )}\}$. Now, let us define the function $\varphi$ as follows. Denote by ${\mathbb {F}}_{q}^{*}$ the multiplicative group of the field ${\mathbb {F}}_{q}$.

$$\begin{aligned} \varphi : {\mathbb {F}}_{q}^{*} \times \left\{ \rho ^{(0)},\ldots ,\rho ^{(\alpha )} \right\} \rightarrow \left\{ \rho ^{(0)},\ldots ,\rho ^{(\alpha )} \right\} , \end{aligned}$$

where

$$\begin{aligned} \varphi (r,\rho ^{(i)})= \left[ \rho ^{(i)}_{0},\rho ^{(i)}_{\pi _{r(1)}}, \ldots ,\rho ^{(i)}_{\pi _{r(s)}}\right] = \rho ^{(j)}, \end{aligned}$$

where

$$\begin{aligned} \pi _{r}(l)=|k| \hbox { such that } rk \equiv l \mod q \hbox { and } -s\le k \le s, \ r\in {\mathbb {F}}_{q}^{*}. \end{aligned}$$

### **Lemma 1**

*The function*$\varphi$*is a group action of*${\mathbb {F}}_{q}^{*}$, *whereqis prime, on the set*$\{\rho ^{(0)},\ldots ,\rho ^{(\alpha )}\}$*of Lee-compositions*.

Let us show that the above function $\varphi$ is in fact a group action. Clearly the identity property is satisfied as

$$\begin{aligned} \varphi (1,\rho ^{(i)})= \left[ \rho ^{(i)}_{0},\rho ^{(i)}_{1}, \ldots ,\rho ^{(i)}_{s}\right] = \rho ^{(i)}. \end{aligned}$$

The compatibility property requires that $\varphi (r_{1},\varphi (r_{2},\rho ^{(i)})) = \varphi (r_{1}\cdot r_{2}, \rho ^{(i)})$, where $r_{1},r_{2}\in {\mathbb {F}}_{q}^{*}$. Let us write

$$\begin{aligned} \varphi (r_{2},\rho ^{(i)})= \left[ \rho ^{(i)}_{0}, \rho ^{(i)}_{\pi _{r_{2}(1)}}, \ldots , \rho ^{(i)}_{\pi _{r_{2}(s)}} \right] . \end{aligned}$$

Then,

$$\begin{aligned} \varphi (r_{1},(\varphi (r_{2},\rho ^{(i)})) = \left[ \rho ^{(i)}_{0}, \rho ^{(i)}_{\pi _{r_{1}\left(\pi _{r_{2}(1)}\right)}},\ldots ,\rho ^{(i)}_{\pi _{r_{1} \left(\pi _{r_{2}(s)}\right)}}\right] . \end{aligned}$$

Now,

$$\begin{aligned} \varphi (r_{1}\cdot r_{2},\rho ^{(i)}) = \left[ \rho ^{(i)}_{0},\rho ^{(i)}_{\pi _{r_{1}\cdot r_{2}(1)}},\ldots ,\rho ^{(i)}_{\pi _{r_{1}\cdot r_{2}(s)}}\right] . \end{aligned}$$

Therefore, we need to show that $\pi _{r_{1}(\pi _{r_{2}(l)})}= \pi _{r_{1}\cdot r_{2}(l)}$.

Now,

$$\begin{aligned} {\left\{ \begin{array}{ll} \pi _{r_{2}}(l)=|k_{1}| &{}\quad \hbox {such that } r_{2}k_{1} \equiv l \mod q, \ -s\le k_{1}\le s,\\ \pi _{r_{1}}(\pi _{r_{2}}(l))=|k_{2}| &{}\quad \hbox {such that } r_{1}k_{2} \equiv |k_{1}| \mod q, \ -s\le k_{2}\le s,\\ \pi _{r_{1}r_{2}}(l)=|k_{3}| &{}\quad \hbox {such that } r_{1}r_{2}k_{3} \equiv l \mod q, \ -s\le k_{3}\le s. \end{array}\right. } \end{aligned}$$

We need to prove that $|k_{2}|=|k_{3}|$.

### *Proof*

We have two cases. If $1\le k_{1} \le s$ we have

$$\begin{aligned} r_{1}k_{2}\equiv k_{1} \mod q \Rightarrow r_{2}k_{1}r_{1}k_{2}\equiv k_{1}l \mod q \Rightarrow r_{2}r_{1}k_{2}\equiv l \mod q, \end{aligned}$$

and since *q* is prime and $-s\le k_{2},k_{3}\le s$ it must be that $k_{2}=k_{3}$.

If $-s\le k_{1} <0$ we have

$$\begin{aligned} r_{1}k_{2}\equiv -k_{1} \mod q \Rightarrow r_{2}k_{1}r_{1}k_{2}\equiv -k_{1}l \mod q \Rightarrow r_{2}r_{1}(-k_{2})\equiv l \mod q, \end{aligned}$$

and since *q* is prime and $-s\le k_{2},k_{3}\le s$ it must be that $|k_{2}|=|k_{3}|$. $\square$

The action of ${\mathbb {F}}_{q}^{*}$ on the set of Lee-compositions partitions the set into equivalence classes, which are called orbits. So, the orbit of an element $\rho ^{(i)}$ is

$$\begin{aligned} Orb(\rho ^{(i)}) = \left\{ \varphi \left( r,\rho ^{(i)}\right) : r\in {\mathbb {F}}_{q}^{*} \right\} . \end{aligned}$$

Clearly there is a correspondence between $\tau ({\mathbf {t}}_{i})$ and the orbits $Orb(\rho ^{(i)})$, i.e., the sets $\tau ({\mathbf {t}})$ are the orbits of the above group action. Therefore, the theory of group actions can be used for studying the Lee-compositions and the linear programming problem, e.g., for the compact problem that we introduce in this paper, the orbit-counting theorem (see, for instance, Burnside 1897) can be used for determining the complexity of the problem as the number of orbits equals the number of variables in the compact problem.

## Properties of certain sums of Lee-numbers

In this section we study certain sums of Lee-numbers, where the Lee-numbers are taken over Lee-compositions belonging to a given orbit. We show that this type of a sum is rational as opposed to the Lee-numbers, which in many cases are irrational numbers, and that it satisfies certain equality constraints.

Since the values of the coefficients of the inner distribution are equal for all compositions in $\tau ({\mathbf {t}}_{i})$, we will have in (5) sums of the form

$$\begin{aligned} B_{{\mathbf {t}}_{i}}\cdot \left( L_{\mathbf {k}} ({\mathbf {t}}_{i_{1}}) + L_{\mathbf {k}}({\mathbf {t}}_{i_{2}}) +\cdots + L_{\mathbf {k}}({\mathbf {t}}_{i_{u}}) \right) . \end{aligned}$$

Therefore, for each coefficient $B_{{\mathbf {t}}_{i}}$ we are computing a sum of Lee-numbers, where the Lee-numbers are taken over the Lee-compositions belonging to $\tau ({\mathbf {t}}_{i})$.

Let us introduce the following lemma.

### **Lemma 2**

*Let*$\tau ({\mathbf {t}}_{i})= \{{\mathbf {t}}_{i_{1}}, \ldots ,{\mathbf {t}}_{i_{u}}\}$. *Then the sum*

6 $$\begin{aligned} L_{\mathbf {k}}({\mathbf {t}}_{i_{1}})+L_{\mathbf {k}} ({\mathbf {t}}_{i_{2}})+\cdots +L_{\mathbf {k}}({\mathbf {t}}_{i_{u}}), \end{aligned}$$

*is a rational number*.

### *Proof*

First, we want to change each term $L_{\mathbf {k}}({\mathbf {t}}_{i})$ into $L_{{\mathbf {t}}_{i}}({\mathbf {k}})$. There is the following relationship between the Lee-numbers $L_{\mathbf {k}}(\mathbf {t})$ and $L_{\mathbf {t}}(\mathbf {k})$ (see Astola 1982b):

7 $$\begin{aligned} \left[ \begin{matrix} n \\ {\mathbf {t}}\end{matrix}\right] L_{\mathbf {k}}({\mathbf {t}})=\left[ \begin{matrix} n \\ {\mathbf {k}} \end{matrix}\right] L_{\mathbf {t}}({\mathbf {k}}), \end{aligned}$$

where the coefficient $\genfrac[]{0.0pt}{}{n}{\mathbf {t}}$ corresponds to the number of vectors in ${\mathbb {F}}_{q}^{n}$ for which the Lee-composition is $\mathbf {t}$ (see Astola 1982b). Hence, we can write (6) as

$$\begin{aligned} \left[ \begin{matrix} n \\ {\mathbf {k}} \end{matrix}\right] \big /\left[ \begin{matrix} n \\ {\mathbf {t}}_{i_{1}} \end{matrix}\right] L_{{\mathbf {t}}_{i_{1}}}({\mathbf {k}})+\left[ \begin{matrix} n\\ {\mathbf {k}}\end{matrix}\right] \big /\left[ \begin{matrix} n \\ {\mathbf {t}}_{i_{2}} \end{matrix}\right] L_{{\mathbf {t}}_{i_{2}}}({\mathbf {k}})+\cdots +\left[ \begin{matrix} n \\ {\mathbf {k}}\end{matrix}\right] \big /\left[ \begin{matrix} n \\ {\mathbf {t}}_{i_{u}}\end{matrix}\right] L_{{\mathbf {t}}_{i_{u}}}({\mathbf {k}}). \end{aligned}$$

Now, we notice that since all ${\mathbf {t}}_{i}$ belong to the same $\tau$, they are permutations of each other. This means that the coefficients $\genfrac[]{0.0pt}{}{n}{{\mathbf {t}}_{i}}$ are all equal and (6) takes the form

$$\begin{aligned} \left[ \begin{matrix} n \\ {\mathbf {k}}\end{matrix}\right] \big /\left[ \begin{matrix} n \\ {\mathbf {t}}_{i_{1}} \end{matrix}\right] \left( L_{{\mathbf {t}}_{i_{1}}}({\mathbf {k}}) + L_{{\mathbf {t}}_{i_{2}}}({\mathbf {k}})+ \cdots +L_{{\mathbf {t}}_{i_{u}}}({\mathbf {k}})\right) , \end{aligned}$$

where $\genfrac[]{0.0pt}{}{n}{{\mathbf {k}}}/\genfrac[]{0.0pt}{}{n}{{\mathbf {t}}_{i_{1}}}= 1$ if ${\mathbf {k}}\in \tau ({\mathbf {t}}_{i})$, and a rational number otherwise.

Now, using Eq. (1), we can write the sum in parentheses above as

8 $$\begin{aligned}&L_{{\mathbf {t}}_{i_{1}}}({\mathbf {k}})+L_{{\mathbf {t}}_{i_{2}}} ({\mathbf {k}})+ \cdots +L_{{\mathbf {t}}_{i_{u}}}({\mathbf {k}}) \nonumber \\&\quad = \sum _{{\mathbf {x}}\mid l({\mathbf {x}})={\mathbf {t}}_{i_{1}}}\left( \prod _{i=1}^{n}\xi ^{v_{i}x_{i}}\right) + \sum _{{\mathbf {x}}\mid l({\mathbf {x}})={\mathbf {t}}_{i_{2}}} \left( \prod _{i=1}^{n}\xi ^{v_{i}x_{i}}\right) +\cdots + \sum _{{\mathbf {x}}\mid l({\mathbf {x}})={\mathbf {t}}_{i_{u}}} \left( \prod _{i=1}^{n}\xi ^{v_{i}x_{i}}\right) \end{aligned}$$

where *u* is the cardinality of $\tau ({\mathbf {t}}_{i})$ and $l({\mathbf {v}})={\mathbf {k}}$.

Since the Lee-numbers $L_{{\mathbf {t}}_{i}}({\mathbf {k}})$ correspond to compositions according to $\tau$, then for each vector ${\mathbf {x}}$, the sum in (8) includes all the vectors $r{\mathbf {x}}$, where $r\in \{1,\ldots ,q-1\}$. Also, each vector can only have one Lee-composition and thus can appear in only one of the sums in (8).

We may now rearrange and group the terms in (8) as

$$\begin{aligned} \left( \xi ^{{\mathbf {v}}\cdot {\mathbf {x}}_{1}}+\xi ^{{\mathbf {v}}\cdot 2{\mathbf {x}}_{1}}+ \cdots + \xi ^{{\mathbf {v}}\cdot (q-1){\mathbf {x}}_{1}}\right) + \left( \xi ^{{\mathbf {v}}\cdot {\mathbf {x}}_{2}}+\xi ^{{\mathbf {v}}\cdot 2{\mathbf {x}}_{2}}+ \cdots + \xi ^{{\mathbf {v}}\cdot (q-1){\mathbf {x}}_{2}}\right) +\cdots \end{aligned}$$

This forms a partition of the set of vectors having a Lee-composition in $\tau ({\mathbf {t}}_{i})$, since the relation ${\mathcal {R}}$ defined as $({\mathbf {x}},{\mathbf {y}})\in {\mathcal {R}}_{x}$ iff ${\mathbf {x}}=r{\mathbf {y}}$, $r\in \{1,\ldots ,q-1\}$ is clearly an equivalence relation.

Therefore, we can group the sum into *m* parts, each having $q-1$ terms. We now take one such part:

$$\begin{aligned} \xi ^{{\mathbf {v}}\cdot {\mathbf {x}}_{i}}+\xi ^{{\mathbf {v}}\cdot 2{\mathbf {x}}_{i}}+ \cdots + \xi ^{{\mathbf {v}}\cdot (q-1){\mathbf {x}}_{i}}, \end{aligned}$$

and write it as

$$\begin{aligned} \xi ^{{\mathbf {v}}\cdot {\mathbf {x}}_{i}}+\xi ^{2({\mathbf {v}}\cdot {\mathbf {x}}_{i})}+ \cdots + \xi ^{(q-1)({\mathbf {v}}\cdot {\mathbf {x}}_{i})}. \end{aligned}$$

If ${\mathbf {v}}\cdot {\mathbf {x}}_{i}=0$ it equals $q-1$, otherwise, we have a sum of the form

$$\begin{aligned} \xi +\xi ^{2}+ \cdots + \xi ^{q-1}=-1. \end{aligned}$$

So (8) is a sum of the form $m_{1}(q-1)+m_{2}(-1)$, where $m_{1}$ and $m_{2}$ are integers $\ge 0$ such that $m=m_{1}+m_{2}$. $\square$

Furthermore, we notice that when we look at the sums

9 $$\begin{aligned} \begin{matrix} L_{{\mathbf {k}}_{i_{1}}}({\mathbf {t}}_{i_{1}})+L_{{\mathbf {k}}_{i_{1}}} ({\mathbf {t}}_{i_{2}})+\cdots +L_{{\mathbf {k}}_{i_{1}}} ({\mathbf {t}}_{i_{u}}), \\ L_{{\mathbf {k}}_{i_{2}}}({\mathbf {t}}_{i_{1}})+L_{{\mathbf {k}}_{i_{2}}} ({\mathbf {t}}_{i_{2}})+\cdots +L_{{\mathbf {k}}_{i_{2}}}({\mathbf {t}}_{{i_{u}}}),\\ \vdots \\ L_{{\mathbf {k}}_{i_{u'}}}({\mathbf {t}}_{i_{1}})+L_{{\mathbf {k}}_{i_{u'}}} ({\mathbf {t}}_{i_{2}})+\cdots +L_{{\mathbf {k}}_{i_{u'}}}({\mathbf {t}}_{i_{u}}), \end{matrix} \end{aligned}$$

where *u* is the cardinality of $\tau ({\mathbf {t}}_{i})$ and $u'$ is the cardinality of $\tau ({\mathbf {k}}_{i})$, they all turn out be equal.

### **Lemma 3**

*For*$${\mathbf {t}}_{i_{1}},\ldots ,{\mathbf {t}}_{i_{u}}\in \tau ({\mathbf {t}}_{i})$$*and*$${\mathbf {k}}_{i},{\mathbf {k}}_{i'}\in \tau ({\mathbf {k}}_{i})$$,

$$\begin{aligned} L_{{\mathbf {k}}_{i}}({\mathbf {t}}_{i_{1}})+L_{{\mathbf {k}}_{i}} ({\mathbf {t}}_{i_{2}})+\cdots +L_{{\mathbf {k}}_{i}}({\mathbf {t}}_{i_{u}})=L_{{\mathbf {k}}_{i'}} ({\mathbf {t}}_{i_{1}})+L_{{\mathbf {k}}_{i'}}({\mathbf {t}}_{i_{2}})+\cdots +L_{{\mathbf {k}}_{i'}}({\mathbf {t}}_{i_{u}}). \end{aligned}$$

### *Proof*

First, we transform these sums using (7) into sums of the following form

$$\begin{aligned}&\left[ \begin{matrix} n \\ {\mathbf {k}}_{i} \end{matrix}\right] \big /\left[ \begin{matrix} n \\ {\mathbf {t}}_{i_{1}} \end{matrix}\right] \left( L_{{\mathbf {t}}_{i_{1}}}({\mathbf {k}}_{i})+L_{{\mathbf {t}}_{i_{2}}} ({\mathbf {k}}_{i})+\cdots +L_{{\mathbf {t}}_{i_{u}}}({\mathbf {k}}_{i})\right) , \\&\left[ \begin{matrix} n \\ {\mathbf {k}}_{i'} \end{matrix}\right] \big /\left[ \begin{matrix} n \\ {\mathbf {t}}_{i_{1}}\end{matrix}\right] \left( L_{{\mathbf {t}}_{i_{1}}} ({\mathbf {k}}_{i'})+L_{{\mathbf {t}}_{i_{2}}}({\mathbf {k}}_{i'})+\cdots +L_{{\mathbf {t}}_{i_{u}}}({\mathbf {k}}_{i'})\right) . \end{aligned}$$

Now we notice that since the Lee-compositions ${\mathbf {k}}_{i}$ and ${\mathbf {k}}_{i'}$ both belong to $\tau ({\mathbf {k}}_{i})$, the coefficients in front of the sums are all equal. What remains to show is that the sums $(L_{{\mathbf {t}}_{i_{1}}} ({\mathbf {k}}_{i})+L_{{\mathbf {t}}_{i_{2}}}({\mathbf {k}}_{i})+\cdots +L_{{\mathbf {t}}_{i_{u}}} ({\mathbf {k}}_{i}))$ and $(L_{{\mathbf {t}}_{i_{1}}}({\mathbf {k}}_{i'}) + L_{{\mathbf {t}}_{i_{2}}}({\mathbf {k}}_{i'})+\cdots +L_{{\mathbf {t}}_{i_{u}}}({\mathbf {k}}_{i'}))$ are equal. To show this, we rearrange both sums as we did in the previous proof to obtain sums of the following form. For the first sum we have

$$\begin{aligned} \left( \xi ^{{\mathbf {v}}\cdot {\mathbf {x}}_{1}}+\xi ^{2({\mathbf {v}}\cdot {\mathbf {x}}_{1})}+ \cdots + \xi ^{(q-1){\mathbf {v}}\cdot {\mathbf {x}}_{1}}\right) + \left( \xi ^{{\mathbf {v}}\cdot {\mathbf {x}}_{2}} + \xi ^{2({\mathbf {v}}\cdot {\mathbf {x}}_{2})}+ \cdots + \xi ^{(q-1){\mathbf {v}}\cdot {\mathbf {x}}_{2}}\right) +\cdots , \end{aligned}$$

where $$l({\mathbf {v}})={\mathbf {k}}_{i}$$ and $$l({\mathbf {x}}_{i}) = {\mathbf {t}}_{i}$$.

Now, since the Lee-compositions ${\mathbf {k}}_{i}$ and ${\mathbf {k}}_{i'}$ belong to $\tau ({\mathbf {k}}_{i})$, we obtain the vectors having the Lee-composition ${\mathbf {k}}_{i'}$ from the vectors having the Lee-composition ${\mathbf {k}}_{i}$ by multiplication by some *r*. Therefore, for the second sum we have

$$\begin{aligned} \left( \xi ^{r{\mathbf {v}}\cdot {\mathbf {x}}_{1}}+\xi ^{r2({\mathbf {v}}\cdot {\mathbf {x}}_{1})}+ \cdots + \xi ^{r(q-1){\mathbf {v}}\cdot {\mathbf {x}}_{1}}\right) + \left( \xi ^{r{\mathbf {v}} \cdot {\mathbf {x}}_{2}}+\xi ^{r2({\mathbf {v}}\cdot {\mathbf {x}}_{2})}+ \cdots + \xi ^{r(q-1){\mathbf {v}}\cdot {\mathbf {x}}_{2}}\right) +\cdots . \end{aligned}$$

Now

$$\begin{aligned} \xi ^{{\mathbf {v}}\cdot {\mathbf {x}}_{i}}+\xi ^{2({\mathbf {v}}\cdot {\mathbf {x}}_{i})}+ \cdots + \xi ^{(q-1){\mathbf {v}}\cdot {\mathbf {x}}_{i}}=\xi ^{r{\mathbf {v}}\cdot {\mathbf {x}}_{i}} + \xi ^{r2({\mathbf {v}}\cdot {\mathbf {x}}_{i})}+ \cdots + \xi ^{r(q-1){\mathbf {v}}\cdot {\mathbf {x}}_{i}}, \end{aligned}$$

since if ${\mathbf {v}}\cdot {\mathbf {x}}_{i}=0$, they are both equal to $q-1$, and otherwise each exponent is distinct. Therefore, the sums are equal. $\square$

## The compact linear programming problem

The additional equalities for linear Lee codes in (4) can be used for compacting the set of linear constraints in the linear programming problem of linear Lee codes. We enforce (3) by replacing all variables $B_{{\mathbf {t}}_{j}}$ for ${\mathbf {t}}_{j}\in \tau ({\mathbf {t}}_{i})$ with a single variable $\gamma _{i}$, so that $\gamma _{i}=B_{{\mathbf {t}}_{j}}$ for all ${\mathbf {t}}_{j}\in \tau ({\mathbf {t}}_{i})$.

We notice that by replacing the $\alpha$ variables $B_{{\mathbf {t}}_1},\ldots ,B_{{\mathbf {t}}_\alpha }$ of the linear programming problem with the set of variables $\gamma _1,\ldots ,\gamma _{\kappa }$, where $\kappa$ is the number of orbits, i.e., the number of different sets $\tau _{i}$, we are eliminating the equality constraints from the sharpened linear programming problem. Let us denote by $\Upsilon$ a matrix, where we have listed all the Lee-numbers as

$$\begin{aligned} \Upsilon = \left[ \begin{matrix} L_{\mathbf {k_{0}}}(\mathbf {t_{0}}) &{} L_{\mathbf {k_{0}}}({\mathbf {t_{1}}}) &{} \cdots &{} L_{\mathbf {k_{0}}}({\mathbf {t_{\alpha }}})\\ L_{\mathbf {k_{1}}}({\mathbf {t_{0}}}) &{} L_{\mathbf {k_{1}}}({\mathbf {t_{1}}}) &{} \cdots &{} L_{\mathbf {k_{1}}}({\mathbf {t_{\alpha }}})\\ \vdots &{} &{} \ddots &{} \vdots \\ L_{\mathbf {k_{\alpha }}}({\mathbf {t_{0}}}) &{} L_{\mathbf {k_{\alpha }}}({\mathbf {t_{1}}}) &{} \cdots &{} L_{\mathbf {k_{\alpha }}}({\mathbf {t_{\alpha }}})\end{matrix}\right] \end{aligned}$$

Let $\mathbf {A}$ be an $\alpha \times \kappa$ matrix of the form

$$\begin{aligned} {\mathbf {A}} = \left[ \begin{matrix}1 &{} 0 &{} 0 &{} 0 &{} \cdots &{} 0\\ \hline 0 &{} 1 &{} 0 &{} 0 &{} \cdots &{} 0\\ 0 &{} 1 &{} 0 &{} 0 &{} \cdots &{} 0\\ 0 &{} 1 &{} 0 &{} 0 &{} \cdots &{} 0\\ 0 &{} 1 &{} 0 &{} 0 &{} \cdots &{} 0 \\ \hline 0 &{} 0 &{} 1 &{} 0 &{} \cdots &{} 0 \\ 0 &{} 0 &{} 1 &{} 0 &{} \cdots &{} 0 \\ \hline \cdots \\ \hline 0 &{} 0 &{} 0 &{} 0 &{} \cdots &{} 1 \end{matrix}\right] , \end{aligned}$$

where the number of rows inside each partition corresponds to the cardinalities of the sets $\tau _{i}$.

We introduce the vector $\varvec{\gamma } =(\gamma _0,\gamma _1,\ldots ,\gamma _\kappa )$ and formulate the linear programming problem equivalent to (3):

$$\begin{aligned}&\hbox {maximize} \sum _{i=1}^\kappa |\tau _i |\gamma _i \\&\ \ \ \gamma _0 =1 \text{ and } \gamma _i \ge 0, \quad i\in I \text{ and } \gamma _i=0, \quad i\in \{1,\ldots ,\alpha \}{\setminus } I \\&\ \ \ \Upsilon {\mathbf {A}} \gamma \ge 0 , \end{aligned}$$

where $I = \{i\mid \forall {\mathbf {t}}\in \tau _{i} : l({\mathbf {x}})={\mathbf {t}} \Rightarrow w_{L}({\mathbf {x}})\ge d\}$, i.e., the indices of those sets $\tau _{i}$, where all Lee-compositions correspond to vectors of Lee weight $\ge d$.

The cardinalities $|\tau _i |$ appear in the criterion of the problem since the initial criterion expressed in terms of the variables $B_{{\mathbf {t}}_1},\ldots ,B_{{\mathbf {t}}_\alpha }$ is ${\mathbf {1}}^T {\mathbf {B}}$, where $\mathbf {1}$ is the all one vector and ${\mathbf {B}}=[B_{{\mathbf {t}}_1},\ldots ,B_{{\mathbf {t}}_\alpha }]^{T}$, and the criterion in the new variables is ${\mathbf {1}}^T {\mathbf {A}} \varvec{\gamma }$, where the new vector of coefficients, ${\mathbf {1}}^T {\mathbf {A}}$, will have as elements the size of the partitions of ${\mathbf {A}}$, which are equal to the cardinalities of the sets $\tau _i$.

Additionally we notice that the matrix ${\mathcal{U}} = \Upsilon {\mathbf {A}}$ can be seen to have the partition structure similar to that of *A*,

$$\begin{aligned} {\mathcal{U}}& = {} \Upsilon {\mathbf {A}} = \Upsilon \left[ \begin{matrix} 1 &{} 0 &{} 0 &{} 0 &{} \cdots &{} 0\\ \hline 0 &{} 1 &{} 0 &{} 0 &{} \cdots &{} 0\\ 0 &{} 1 &{} 0 &{} 0 &{} \cdots &{} 0\\ 0 &{} 1 &{} 0 &{} 0 &{} \cdots &{} 0\\ 0 &{} 1 &{} 0 &{} 0 &{} \cdots &{} 0 \\ \hline 0 &{} 0 &{} 1 &{} 0 &{} \cdots &{} 0 \\ 0 &{} 0 &{} 1 &{} 0 &{} \cdots &{} 0 \\ \hline \cdots \\ \hline 0 &{} 0 &{} 0 &{} 0 &{} \cdots &{} 1 \end{matrix}\right] = \left[ \begin{matrix} \Phi _{1,1} &{}\Phi _{1,2} &{} \Phi _{1,3} &{} \Phi _{1,4} &{} \cdots &{} \Phi _{1,\kappa }\\ \hline \Phi _{2,1} &{}\Phi _{2,2} &{} \Phi _{2,3} &{} \Phi _{2,4} &{} \cdots &{} \Phi _{2,\kappa }\\ \Phi _{2,1} &{}\Phi _{2,2} &{} \Phi _{2,3} &{} \Phi _{2,4} &{} \cdots &{} \Phi _{2,\kappa }\\ \Phi _{2,1} &{}\Phi _{2,2} &{} \Phi _{2,3} &{} \Phi _{2,4} &{} \cdots &{} \Phi _{2,\kappa }\\ \Phi _{2,1} &{}\Phi _{2,2} &{} \Phi _{2,3} &{} \Phi _{2,4} &{} \cdots &{} \Phi _{2,\kappa } \\ \hline \Phi _{3,1} &{}\Phi _{3,2} &{} \Phi _{3,3} &{} \Phi _{3,4} &{} \cdots &{} \Phi _{3,\kappa } \\ \Phi _{3,1} &{}\Phi _{3,2} &{} \Phi _{3,3} &{} \Phi _{3,4} &{} \cdots &{} \Phi _{3,\kappa } \\ \hline \cdots \\ \hline \Phi _{\kappa ,1} &{}\Phi _{\kappa ,2} &{} \Phi _{\kappa ,3} &{} \Phi _{\kappa ,4} &{} \cdots &{} \Phi _{\kappa ,\kappa } \end{matrix}\right] \\& = {} {\mathbf {A}} \Phi \end{aligned}$$

where the matrix $\Phi =[\Phi ]_{i,j}$ is $\kappa \times \kappa$ and the rows in a partition correspond to the Lee-compositions belonging to the same set $\tau _i$. Inside a partition the rows of the matrix $\mathcal{U}$ are identical, due to Lemma 3, since the columns of $\mathcal{U}$ inside a partition have the following elements

$$\begin{aligned} \begin{matrix} L_{{\mathbf {k}}_{i_{1}}}({\mathbf {t}}_{i_{1}})+L_{{\mathbf {k}}_{i_{1}}} ({\mathbf {t}}_{i_{2}})+\cdots +L_{{\mathbf {k}}_{i_{1}}}({\mathbf {t}}_{i_{u}}), \\ L_{{\mathbf {k}}_{i_{2}}}({\mathbf {t}}_{i_{1}})+L_{{\mathbf {k}}_{i_{2}}} ({\mathbf {t}}_{i_{2}})+\cdots +L_{{\mathbf {k}}_{i_{2}}}({\mathbf {t}}_{{i_{u}}}),\\ \vdots \\ L_{{\mathbf {k}}_{i_{u'}}}({\mathbf {t}}_{i_{1}})+L_{{\mathbf {k}}_{i_{u'}}} ({\mathbf {t}}_{i_{2}})+\cdots +L_{{\mathbf {k}}_{i_{u'}}}({\mathbf {t}}_{i_{u}}),\end{matrix} \end{aligned}$$

where $|\tau _{{\mathbf {k}}_{i}}|=u'$ and $|\tau _{{\mathbf {t}}_{i}}|=u$. This leads to a repeated inequality constraint. In order to remove this redundancy, we select from the matrix $\mathcal{U}$ only one row per partition block, keeping thus only the non-redundant inequalities. This is just the matrix $\Phi$, and we may replace the inequality constraints $\Upsilon {\mathbf {A}} \varvec{\gamma } \ge 0$ with $\Phi \varvec{\gamma } \ge 0$. In addition, due to Lemma 2, each element of $\Phi$ is a rational number, and the Lee-numbers can be computed recursively with integers using the recursive formula given by Astola and Tabus (2013). Hence, we can perform all computations using integers instead of irrationals.

In Tables 1, 2, 3 and 4 there are numerical results for the linear programming bounds for linear Lee codes with $q=5,7,13,17$. The bounds were computed using the above compact linear programming bound for linear Lee codes and compared to the general linear programming bound given by (2). Italics indicates an improvement and * indicates a tight bound.

**Table 1:** Upper bounds for the dimension *k* of linear Lee codes when $q=5$

n\d	3	4	5	6	7	8	9	10	11	12	13	14	15	16	Time [s]
2	1*														0.007
3	1*	1*													0.013
4	2*	2*	1*	1*											0.026
5	3*	3*	2*	1*	1*										0.041
6	4*	3*	3*	2*	1*	1*	1*								0.068
7	5*	4*	3*	3*	2*	1*	1*	1*							0.102
8	6*	5*	4*	4*	3*	2*	2*	1*	1*	1*					0.169
9	7*	6*	5*	5	4*	3*	3	2*	1*	1*	1*				0.249
10	8*	7*	6*	6	5	4	3*	3	2*	2*	1*	1*	1*		0.408
11	9*	8*	7	6*	6	5	4	4	3	2*	2*	1*	1*	1*	0.563
12	10*	9*	8	7	7	6	5	5	4	3*	2*	2*	2*	1*	
13	10*	10*	9	8	7	7	6	5	5	4	3*	2*	2*	1*	
14	11*	11	10	9	8	8	7	6	*5*	5	4	3*	3	2*	
15	12*	12	11	10	9	9	8	7	6	6	5	4	4	3	

n\d	17	18	19	20	21	22	23	24	25	26	27	28	29	30	Time [s]
12	1*	1*													0.872
13	1*	1*	1*												1.426
14	1*	1*	1*	1*	1*										2.517
15	2*	2*	1*	1*	1*	1*									3.951

The * indicates a tight bound and italics an improvement compared to the bound given by (2)

**Table 2:** Upper bounds for the dimension *k* of linear Lee codes when $q=7$

n\d	3	4	5	6	7	8	9	10	11	12	13	14	15	16	Time [s]
2	1*														0.009
3	2*	1*	1*	1*											0.028
4	2*	2*	2*	1*	1*										0.047
5	3*	3*	2*	2*	1*	1*	1*								0.098
6	4*	4*	3*	3*	2*	2*	1*	1*	1*	1*					0.212
7	5*	5*	4*	4	3*	3	2*	2*	1*	1*	1*				0.420
8	6*	5*	5*	4*	4*	4	3*	3	2*	2*	1*	1*	1*		0.816
9	7*	6*	6	5*	5	4*	4	3*	3	3	2*	1*	1*	1*	
10	8*	7*	7	6*	6	5	5	4	4	3*	3	2*	2*	1*	
11	9*	8*	8	7	6*	*5*	5	5	5	4	4	3	3	2*	
12	10*	9*	8*	8	7	7	6	6	5	5	4	4	3	3	
13	11*	10*	9*	9	8	8	7	7	6	6	5	5	4	4	
14	12*	11*	10*	10	9	9	8	8	7	7	6	6	5	5	
15	13*	12*	11*	11	10	10	9	9	8	*7*	7	6	6	*5*	

n\d	17	18	19	20	21	22	23	24	25	26	27	28	29	30	Time [s]
9	1*	1*													1.621
10	1*	1*	1*												2.959
11	2*	2	1*	1*	1*										6.406
12	3	2*	2	1*	1*	1*	1*	1*							17.580
13	*3*	3	2*	2*	2	1*	1*	1*	1*						31.983
14	4	4	3	3	2*	2*	2	1*	1*	1*	1*				67.885
15	5	5	4	4	3	3	2*	2*	1*	1*	1*	1*	1*	1*	173.448

The * indicates a tight bound and italics an improvement compared to the bound given by (2)

**Table 3:** Upper bounds for the dimension *k* of linear Lee codes when $q=13$

n\d	3	4	5	6	7	8	9	10	11	12	13	14	15	16	Time [s]
2	1*	1*	1*												0.038
3	2*	1*	1*	1*	1*	1*	1								0.107
4	3*	2*	2*	2*	2*	1*	1*	1*	1*	1*	1				0.487
5	4*	3*	3*	3	2*	2*	2*	2	1*	1*	1*	1*	1*	1	
6	5*	4*	4*	3*	3*	3*	3	2*	2*	2*	2	1*	1*	1*	
7	5*	5*	5	4*	4*	4	3*	3*	3*	3	2*	2*	2*	2*	
8	6*	6*	6	5*	5	5	4*	4	4	3*	3*	3	3	2*	

n\d	17	18	19	20	21	22	23	24	25	26	27	28	29	30	Time [s]
5	1														2.288
6	1*	1*	1*	1*	1*										11.508
7	2	1*	1*	1*	1*	1*									57.325
8	2*	2*	2*	2	2	1*	1*	1*	1*	1*					300.543

The * indicates a tight bound and italics an improvement compared to the bound given by (2)

**Table 4:** Upper bounds for the dimension *k* of linear Lee codes when $q=17$

n\d	3	4	5	6	7	8	9	10	11	12	13	14	15	16	Time [s]
2	1*	1*	1*												0.043
3	2*	2*	1*	1*	1*	1*	1*								0.276
4	3*	2*	2*	2*	2*	2*	1*	1*	1*	1*	1*	1*	1*		2.271
5	4*	3*	3*	3*	2*	2*	2*	2*	2*	2	1*	1*	1*	1*	
6	5*	4*	4*	4*	3*	3*	3*	3	2*	2*	2*	2*	2*	2*	

n\d	17	18	19	20	21	22	23	24	25	26	27	28	29	30	Time [s]
5	1*	1*	1*												19.117
6	1*	1*	1*	1*	1*	1*	1*	1*							188.785

The $*$ indicates a tight bound and italics an improvement compared to the bound given by (2)

In Tables 1, 2, 3 and 4, the computation time is also shown for each *n*. When computing the sharpened linear programming bound by giving additional equality constraints to the linear programming solver, more computation time is required for given parameters than with the general linear programming problem given by (2), since the linear programming solver is given a larger set of overall constraints. However, using the compact problem introduced in this paper reduces the computation time significantly, since there are less variables and constraints and all computations can be performed with rational numbers. For example, on an Apple iMac 5K (late 2014) with Intel Core i7 (I7-4790K), 32 gigabytes of memory, and a 64-bit operating system OS X Yosemite, and using the linprog linear programming solver of Matlab, for $q=17$ and $n=5$, computing the regular linear programming bounds took approximately 17 min and computing the sharpened bounds by using additional equality constraints took over 5 h. When using the compact problem, the computation time was less than 20 s, including computation of the new sets of constraints from the Lee-numbers according to the sets $\tau ({\mathbf {t}})$, which is a significant improvement and makes it possible to compute the bounds for larger parameter values in a reasonable time. The Lee-numbers were computed separately, and computing them recursively for $q=17$ and $n=2,\ldots ,6$ took approximately 1 min.

The obtained bounds can be used for identifying optimal codes. Consider the bound for *k* given in Table 4 with $q=17$, $n=5$ and $d=7$, which is 2. This means that the maximum number of codewords in a linear code with these parameters is at most 289. The code having the generator matrix

$$\begin{aligned} {\mathbf {G}}=\left[ \begin{array}{cc|ccc} 1&{}0&{}5&{}0&{}4\\ 0&{}1&{}16&{}15&{}10\end{array}\right] \end{aligned}$$

is a [5, 2]-code with the minimum distance 7 and has $17^2=289$ codewords, therefore, it is an optimal linear code for the above parameters.

## Conclusions

In this paper, we formulated the invariance-type property of Lee-compositions introduced by Astola and Tabus (2015) in terms of an action of the multiplicative group of the field ${\mathbb {F}}_{q}$ on the set of Lee-compositions. This formulation is useful in the theoretical study of Lee-compositions and linear Lee codes. In addition, we have shown some useful properties of certain sums of Lee-numbers that appear in the constraints of the linear programming problem of linear Lee codes. Based on the equality constraints and these properties, we constructed a more compact linear programming problem for linear Lee codes, leading to a fast execution and allowing all computations to be performed using integers. This leads in practice to having available upper bounds for codes with parameters higher than available up to now.
